# Exploration of static functional connectivity and dynamic functional connectivity alterations in the primary visual cortex among patients with high myopia *via* seed-based functional connectivity analysis

**DOI:** 10.3389/fnins.2023.1126262

**Published:** 2023-02-02

**Authors:** Yu Ji, Shui-qin Huang, Qi Cheng, Wen-wen Fu, Pei-pei Zhong, Xiao-lin Chen, Ben-liang Shu, Bin Wei, Qin-yi Huang, Xiao-rong Wu

**Affiliations:** Department of Ophthalmology, The First Affiliated Hospital of Nanchang University, Nanchang, Jiangxi, China

**Keywords:** high myopia, brain function, brain region, resting-state functional magnetic resonance imaging, static functional connectivity, dynamic functional connectivity, seed-based functional connectivity analysis

## Abstract

**Aim:**

This study was conducted to explore differences in static functional connectivity (sFC) and dynamic functional connectivity (dFC) alteration patterns in the primary visual area (V1) among high myopia (HM) patients and healthy controls (HCs) *via* seed-based functional connectivity (FC) analysis.

**Methods:**

Resting-state functional magnetic resonance imaging (fMRI) scans were performed on 82 HM patients and 59 HCs who were closely matched for age, sex, and weight. Seed-based FC analysis was performed to identify alterations in the sFC and dFC patterns of the V1 in HM patients and HCs. Associations between mean sFC and dFC signal values and clinical symptoms in distinct brain areas among HM patients were identified *via* correlation analysis. Static and dynamic changes in brain activity in HM patients were investigated by assessments of sFC and dFC *via* calculation of the total time series mean and sliding-window analysis.

**Results:**

In the left anterior cingulate gyrus (L-ACG)/left superior parietal gyrus (L-SPG) and left V1, sFC values were significantly greater in HM patients than in HCs. In the L-ACG and right V1, sFC values were also significantly greater in HM patients than in HCs [two-tailed, voxel-level *P* < 0.01, Gaussian random field (GRF) correction, cluster-level *P* < 0.05]. In the left calcarine cortex (L-CAL) and left V1, dFC values were significantly lower in HM patients than in HCs. In the right lingual gyrus (R-LING) and right V1, dFC values were also significantly lower in HM patients than in HCs (two-tailed, voxel-level *P* < 0.01, GRF correction, cluster-level *P* < 0.05).

**Conclusion:**

Patients with HM exhibited significantly disturbed FC between the V1 and various brain regions, including L-ACG, L-SPG, L-CAL, and R-LING. This disturbance suggests that patients with HM could exhibit impaired cognitive and emotional processing functions, top-down control of visual attention, and visual information processing functions. HM patients and HCs could be distinguished from each other with high accuracy using sFC and dFC variabilities. These findings may help to identify the neural mechanism of decreased visual performance in HM patients.

## 1. Introduction

High myopia (HM) is a type of ametropia characterized by a need for more than −6.00 diopters of refractive correction or the presence of an axial length ≥ 26 mm^1^ (Ucak et al., 2020). The typical clinical manifestations of HM include decline in distance vision, normal near vision, and decline in dark adaptive function; these manifestations are often accompanied by visual fatigue. Patients with HM can exhibit degenerative changes in the fundus related to excessive elongation of the ocular axis; such changes include patchy or diffuse chorioretinal atrophy, retinal nerve fiber layer thinning, macular atrophy, macular hole formation, and altered retinal vessel morphology (Li et al., 2011; Silva, 2012; Kamal Salah et al., 2015). In East Asia, 80–90% of 18-year-olds exhibit myopia, and approximately 10–20% of these individuals have HM (Jonas and Panda-Jonas, 2019). The incidence rate of HM among Chinese students in primary school does not exceed 1%, but it is > 2% in junior high school (Wang et al., 2018). There are multiple risk factors for increased axial length in patients with HM, such as sex, best-corrected visual acuity, axial length, type of myopic maculopathy, and choroidal neovascularization status (Du et al., 2021). Jonas et al. (2020) demonstrated that the prevalence of glaucoma-like optic neuropathy increases with longer axial length in eyes with HM; other investigations have shown that glaucoma is both an eye disease and a degenerative illness of the central nervous system (Nucci et al., 2015; Mancino et al., 2018). Therefore, a connection may exist between HM and the central nervous system.

Recently, resting-state functional magnetic resonance imaging (fMRI) has emerged as an important method for non-invasive analyses of changes in brain function; it has been used to investigate various clinical diseases. Resting-state fMRI is based on the paramagnetic effect of deoxygenated hemoglobin. Increased oxygen consumption in local tissues leads to an increase in deoxygenated hemoglobin, which indirectly indicates the degree of local neural activity in the brain (Glover, 2011). Patients with HM reportedly exhibit significantly decreased voxel-mirror homotopic connectivity between the putamen and fusiform gyrus, suggesting that the visual and recognition functions are affected in HM patients (Cheng et al., 2022). Huang et al. (2018a) revealed that the whole-brain gray matter volume of the right cuneus gyrus was decreased in patients with HM, indicating potential visual cortex functional impairment. Our previous study demonstrated that HM patients had altered dynamic regional homogeneity values in the left fusiform gyrus, right inferior temporal gyrus, right Rolandic operculum, right postcentral gyrus, and right precentral gyrus (Ji et al., 2022). Thus far, research has mainly focused on changes in static brain activity in HM patients; and it is believed that the functional interaction of brain regions remains unchanged in time during the whole MRI scan, which is obviously not objective. Recent research has revealed time-dependent characteristics of brain activity; even when fully at rest, the brain experiences brief spontaneous oscillations that are strongly associated with its activities. In some respects, a larger value may indicate a higher degree of adaptability (Vincent et al., 2007; Allen et al., 2014). Therefore, dynamic functional connectivity (dFC) could serve as a new indicator of complex brain functional structure through the acquisition of time-dependent connections over short durations of time (Du et al., 2018). Additionally, Preti et al. (2017) found that resting-state functional connectivity (FC) dynamically changed throughout the scan cycle. The above findings suggest that analyses of changes in brain activity in HM patients are not sufficiently comprehensive when conducted solely on the basis of static functional connectivity (sFC) or dFC. To our knowledge, previous studies have not combined static and dynamic analysis methods to characterize altered brain function in HM patients. We suspect that such analyses can be used to improve the broader understanding of altered neural mechanisms in HM patients.

The primary visual area (V1; Brodmann 17), located around the calcarine cortex of the occipital lobe (Ding et al., 2013), is the main source of feed forward visual stimuli in higher-level visual cortices. The lateral geniculate nucleus of the thalamus receives visual stimuli from the retina and sends it to the V1 (Mock et al., 2018). Recent studies have revealed two pathways of visual transmission (ventral and dorsal), both of which originate from the retina and project to the V1 (Yamasaki and Tobimatsu, 2018). The ventral pathway is mainly involved in the perception of shape and color, whereas the dorsal pathway is mainly involved in the perception of motion (Tobimatsu and Celesia, 2006). Visual stimuli received by the V1 are then projected to the secondary visual cortex (V2), which is involved in the perception of color and orientation (Tootell et al., 2003). Because the V1 is the first stage of visual information cortex processing visual signals, V1 impairment results in vision loss. Wu et al. (2020) found that the cortical surface thickness of the right V1 was decreased in patients with HM, suggesting that visual and speech functions were affected in those patients. The aforementioned neuroimaging investigations confirmed that patients with HM exhibit unique functional and structural changes in the V1. However, few studies have examined whether individuals with HM exhibit specific altered FC patterns in the V1. Here, we hypothesized that patients with HM would show characteristic alterations in FC patterns in the V1.

## 2. Participants and methods

### 2.1. Participants

From August 2021 to December 2021, 82 HM patients and 59 healthy controls (HCs) were examined in the Department of Ophthalmology at Nanchang University’s First Affiliated Hospital. For each participant, age, sex, and educational background were all met. Individuals with brain illnesses were excluded on the basis of a clinical examination and physical assessment. All eligible individuals were examined in the same clinic and provided written informed consent to participate in the study. All procedures were conducted in accordance with the Declaration of Helsinki, and the study protocol was approved by Nanchang University’s First Affiliated Hospital’s Medical Ethics Committee (Jiangxi Province, China).

The inclusion criteria for HM patients were binocular visual acuity of −6 diopters or worse; corrected visual acuity of better than 1.0; and the completion of MRI-related tests, optical coherence tomography, ultrasonography, and other ophthalmic examinations. The exclusion criteria for HM patients were binocular visual acuity of better than −6 diopters; presence of retinal detachment, maculopathy, choroidal neovascularization, and/or retinal pigment epithelium disease; and/or history of ocular trauma or ophthalmic surgery, neurological diseases, and/or cerebral infarction.

According to age, sex, and educational background, HCs were chosen at random from Nanchang City. The inclusion criteria for HCs were the absence of eye diseases and major illnesses (e.g., neurological illness or cerebral infarction); the presence of uncorrected vision or visual acuity better than 1.0; and the completion of MRI-related tests, optical coherence tomography, ultrasonography, and other ophthalmic examinations.

### 2.2. fMRI data acquisition

All MRI data were obtained using a 3-TeslaTrio magnetic resonance imaging scanning system (Trio Tim, Siemens Medical Systems, Erlangen, Germany). During image acquisition, we asked the participants to close their eyes, minimize movement, and avoid falling asleep. We also asked the participants to use earplugs to reduce the impacts of head movement and machine noise during scanning. The following three-dimensional high-resolution T1-weighted imaging parameters were used in this study: repetition time = 1,900 ms, echo time = 2.26 ms, thickness = 1, no intersection gap, acquisition matrix = 256 × 256, field of view = 240 × 240 mm^2^, and flip angle = 12°.

### 2.3. fMRI data preprocessing analysis

The Statistical Parametric Mapping (SPM12) and Data Processing and Analysis for Brain Imaging (DPABI) toolboxes running on MATLAB 2013b were used for data preprocessing. The following preprocessing steps were performed: (1) conversion of DICOM format to NIFTI format; (2) removal of the first 10 volumes of functional images to eliminate erratic data related to machine initialization; (3) analysis of functional volumes; (4) time correction; (5) head motion correction; (6) spatial normalization; (7) spatial smoothing; and (8) removal of linear data, interference noise, and low-frequency filtering.

### 2.4. Definition of region of interest (ROI)

The ROI for this investigation was defined as the V1 (Brodmann 17). Computation of FC was conducted with the center of the V1 as the seed point. The Montreal Neurological Institute (MNI) coordinates for the right V1 and left V1 were (8, −76, 10) and (−8, −76, 10), respectively (Table 1). The radius of the ROI was set to 6 mm. The mean time course of the ROI was compared with the time courses of all other regions to generate a Pearson correlation coefficient. Fisher’s z-transform analysis was applied to the Pearson correlation coefficient to evaluate data normality and obtain an approximate normal distribution for further statistical analyses.

**TABLE 1:** Montreal Neurological Institute coordinates for region of interest.

Region of interest	X	Y	Z
Right V1 (Brodmann 17)	8	−76	10
Left V1 (Brodmann 17)	−8	−76	10

MNI, Montreal Neurological Institute.

### 2.5. Seed-based FC analysis

We used seed-based FC analysis to identify the FC of the V1. First, we established the sFC analysis parameters. In the DPABI toolbox, we created a sphere with a radius of 6 mm, then calculated Pearson correlation coefficients for each participant’s seed region and all voxels in the brain in sequence, using Fisher’s z-transform analysis to improve normality. The results were used for sFC analysis. Next, we established dFC analysis parameters. In the DPABI toolbox, we used the sliding window method within the “Dynamic and Stability Analyses” module to measure dFC. We extracted the time series signal of the ROI for each participant, then selected a time window with a width of 30 repetition time and a length of 1 repetition time. The Pearson correlation coefficients of the mean time series signals of all voxels in the whole brain were calculated in a sequential manner; this allowed acquisition of the correlation coefficient of the whole-brain voxel sliding window for the ROI of each participant. Finally, the results of multiple sliding window correlation coefficients for each participant were normalized, and FC variability was represented by calculating the standard deviation of the *z*-value for each voxel correlation coefficient. The results were used for dFC analysis.

### 2.6. Statistical analysis

SPSS 8.0 software was used for analyses of aggregated clinical and demographic data. The chi-squared test was used to analyze proportions, whereas independent two-sample *t*-tests were used to assess continuous variables (*P*-values < 0.05 were considered indicative of statistical significance). Using SPM12 software, one-sample *t*-tests were conducted to assess intragroup *z*-value FC patterns, and two-sample *t*-tests were conducted to investigate differences in *z*-value FC patterns between two groups [voxel-level *P* < 0.01, Gaussian random field (GRF) correction, cluster-level *P* < 0.05]. Additionally, Pearson correlation coefficients were used to examine associations between mean FC signal values in various brain locations and clinical characteristics in patients with HM (*P*-values < 0.05 were considered indicative of statistical significance).

## 3. Results

### 3.1. Demographics

This study included 82 HM patients (43 men and 39 women; mean age, 26.53 ± 5.291 years) and 59 HCs (24 men and 35 women; mean age, 25.67 ± 3.102 years). Demographic characteristics are shown in Table 2.

**TABLE 2:** Demographic characteristics of HM patients and HCs.

Characteristic	HM patients	HCs
Men/women	43/39	24/35
Age (years)	26.53 ± 5.291	25.67 ± 3.102

HM, high myopia; HCs, healthy controls.

### 3.2. Group differences in sFC

**FIGURE 1:**
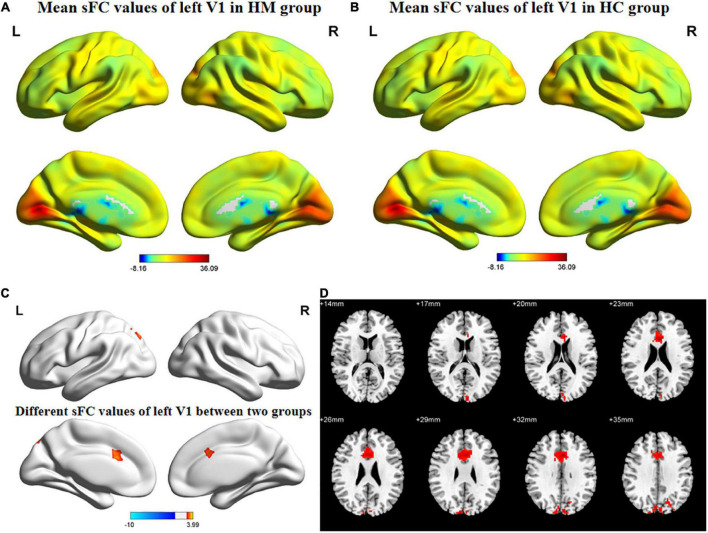
Spatial of distributions of sFC patterns of the left V1 in HM patients and HCs. **(A)** Mean sFC values of left V1 in HM group; **(B)** mean sFC values of left V1 in HC group; **(C)** different sFC values of left V1 between two groups and **(D)** significant zsFC maps of left V1 differences among two groups. HCs, healthy controls; HM, high myopia; sFC, static functional connectivity; zsFC, z-values static functional connectivity; L, left; R, right.

**FIGURE 2:**
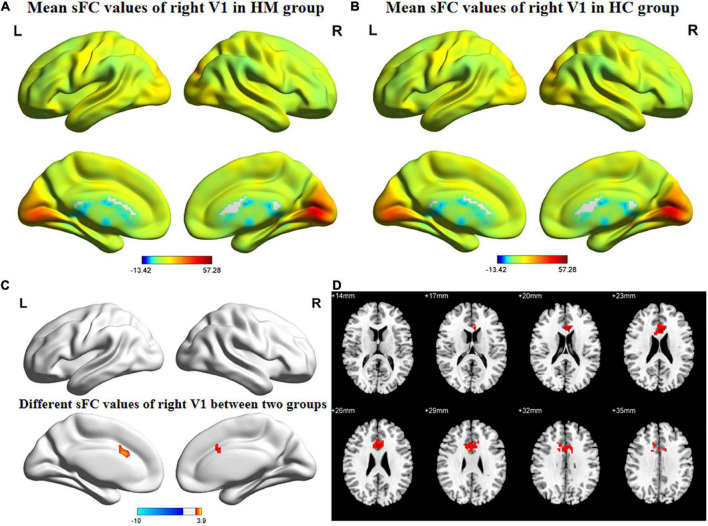
Spatial of distributions of sFC patterns of the right V1 in HM patients and HCs. **(A)** Mean sFC values of right V1 in HM group; **(B)** mean sFC values of right V1 in HC group; **(C)** different sFC values of right V1 between two groups and **(D)** significant zsFC maps of right V1 differences among two groups. HCs, healthy controls; HM, high myopia; sFC, static functional connectivity; zsFC, z-values static functional connectivity; L, left; R, right.

Figures 1, 2 show the spatial distributions of sFC values of the bilateral V1 in HM patients and HCs. In the left anterior cingulate gyrus (L-ACG)/left superior parietal gyrus (L-SPG) and left V1, sFC values were significantly greater in HM patients than in HCs (Figure 1 and Table 3) (voxel-level *P* < 0.01, GRF correction, cluster-level *P* < 0.05). In the L-ACG and right V1, sFC values were also significantly greater in HM patients than in HCs (Figure 2 and Table 4) (voxel-level *P* < 0.01, GRF correction, cluster-level *P* < 0.05).

**TABLE 3:** Significant differences in sFC values of the left V1 between HM patients and HCs.

Brain region	BA	Peak *t*-score	MNI coordinates (x, y, z)	Cluster size (voxels)
L-ACG	32	3.5628	−3, 15, 33	200
L-SPG		3.9879	−18, −81, 48	214

sFC, static functional connectivity; V1, primary visual area; HCs, healthy controls; HM, high myopia; BA, Brodmann area; L-ACG, left anterior cingulate gyrus; L-SPG, left superior parietal gyrus; MNI, Montreal Neurological Institute.

**TABLE 4:** Significant differences in sFC values of the right V1 between HM patients and HCs.

Brain region	BA	Peak *t*-score	MNI coordinates (x, y, z)	Cluster size (voxels)
L-ACG		3.8977	−9, 21, 21	158

sFC, static functional connectivity; V1, primary visual area; HCs, healthy controls; HM, high myopia; BA, Brodmann area; L-ACG, left anterior cingulate gyrus; MNI, Montreal Neurological Institute.

### 3.3. Group differences in dFC

**FIGURE 3:**
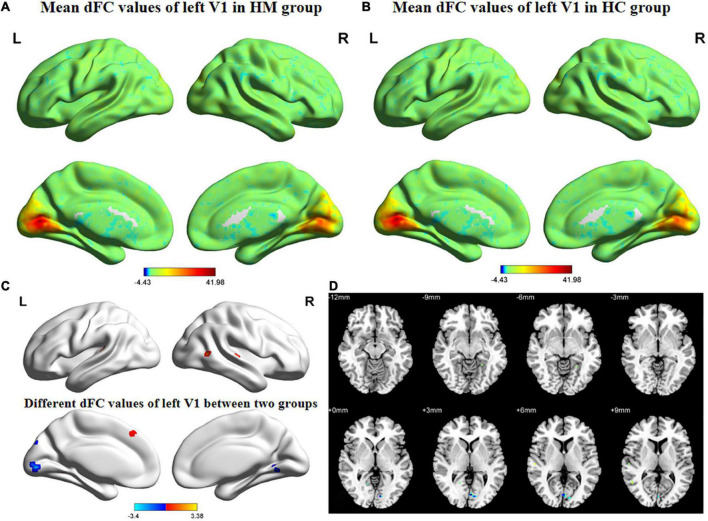
Spatial of distributions of dFC patterns of the left V1 in HM patients and HCs. **(A)** Mean dFC values of left V1 in HM group; **(B)** mean dFC values of left V1 in HC group; **(C)** different dFC values of left V1 between two groups and **(D)** significant zdFC maps of left V1 differences among two groups. HCs, healthy controls; HM, high myopia; sFC, static functional connectivity; zdFC, z-values dynamic functional connectivity; L, left; R, right.

**FIGURE 4:**
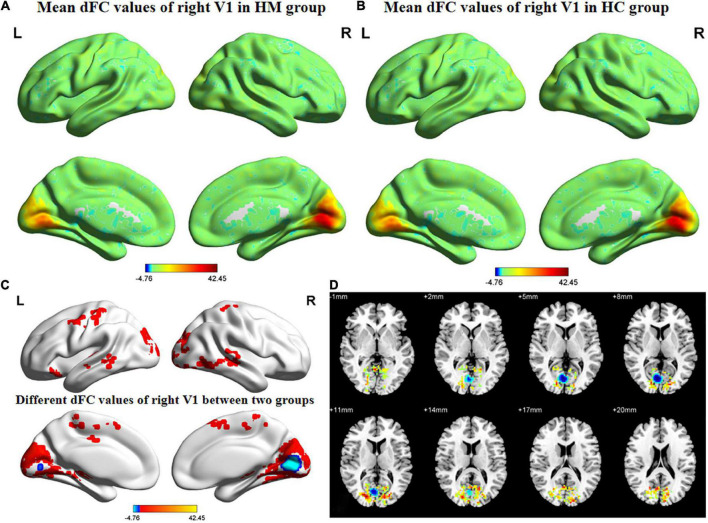
Spatial of distributions of dFC patterns of the right V1 in HM patients and HCs. **(A)** Mean dFC values of right V1 in HM group; **(B)** mean dFC values of right V1 in HC group; **(C)** different dFC values of right V1 between two groups and **(D)** significant zdFC maps of right V1 differences among two groups. HCs, healthy controls; HM, high myopia; sFC, static functional connectivity; zdFC, z-values dynamic functional connectivity; L, left; R, right.

Figures 3, 4 show the spatial distributions of dFC values of the bilateral V1 in HM patients and HCs. In the left calcarine cortex (L-CAL) and left V1, dFC values were significantly lower in HM patients than in HCs (Figure 3 and Table 5) (voxel-level *P* < 0.01, GRF correction, cluster-level *P* < 0.05). In the right lingual gyrus (R-LING) and right V1, dFC values were also significantly lower in HM patients than in HCs (Figure 4 and Table 6) (voxel-level *P* < 0.01, GRF correction, cluster-level *P* < 0.05).

**TABLE 5:** Significant differences in dFC values of the left V1 between HM patients and HCs.

Brain region	BA	Peak *t*-score	MNI coordinates (x, y, z)	Cluster size (voxels)
L-CAL		−3.3452	−3, −81, 3	15

dFC, dynamic functional connectivity; V1, primary visual area; HCs, healthy controls; HM, high myopia; BA, Brodmann area; L-CAL, left calcarine cortex; MNI, Montreal Neurological Institute.

**TABLE 6:** Significant differences in dFC values of the right V1 between HM patients and HCs.

Brain region	BA	Peak t-score	MNI coordinates (x, y, z)	Cluster size (voxels)
R-LING	23	−11.017	6, −75, 9	1,489

dFC, dynamic functional connectivity; V1, primary visual area; HCs, healthy controls; HM, high myopia; BA, Brodmann area; R-LING, right lingual gyrus; MNI, Montreal Neurological Institute.

## 4. Discussion

A reliable and efficient approach to assess the correlation coefficients of blood oxygen level-dependent signal time series between different brain areas and V1 is the seed-based FC technique used in the present study. This method has been used multiple times for analyses of individuals with ophthalmic or other systemic diseases (Table 7). To our knowledge, the present study is the first to investigate the sFC and dFC between the V1 and other brain regions in patients with HM; the findings may facilitate greater understanding of V1 HCs in such patients. Notably, we found that, in the L-ACG/L-SPG and left V1, sFC values were significantly greater in HM patients than in HCs; in the L-ACG and right V1, sFC values were also significantly greater in HM patients than in HCs. However, in the L-CAL and left V1, dFC values were significantly lower in HM patients than in HCs; in the R-LING and right V1, dFC values were also significantly lower in HM patients than in HCs. These results may provide some insights regarding the neural mechanisms involved in HM; they may also be useful in identifying potential neurological causes of decreased visual performance in HM patients.

**TABLE 7:** Use of seed-based FC technique for analysis of individuals with ophthalmic or other systemic diseases.

References	Disease	Year
Wu et al. (2021)	Bronchial asthma	2021
Tang et al. (2021)	Alzheimer’s disease and mild cognitive impairment	2021
Qi et al. (2021)	Diabetic retinopathy	2021
Yu et al. (2020)	Proliferative diabetic retinopathy	2020
Ke et al. (2020)	Migraine without aura	2020
Nie et al. (2015)	Primary insomnia	2015

FC, functional connectivity.

### 4.1. sFC alterations and their significance

Within the bilateral V1 and L-ACG in HM patients, significantly increased sFC was observed. The anterior cingulate gyrus, which plays a key role in regulating cognitive and emotional processing (Takayanagi et al., 2013), is an important anatomical component of the salience network (Christopher et al., 2015). The salience network acts as a mediator within the brain, continuously monitoring the external environment and assessing how other brain networks respond to new information and stimuli. Previous studies have shown that the salience network is primarily responsible for regulating the switch between the default mode network and the central execution network, avoiding simultaneous excitation of both networks (Goulden et al., 2014). Philippi et al. (2018) found that major depressive disorder patients with greater negative self-focus thoughts showed significantly increased sFC within the anterior cingulate gyrus. Hafkemeijer et al. (2013) demonstrated that Subjective memory complaints (SMC) patients had significantly increased sFC values in the anterior cingulate gyrus, implying that the increase in sFC is related to compensation for the loss of cognitive function and maintenance of task performance. Sacca et al. (2022) reported that the treatment of migraine with 20-Hz transcutaneous auricular vagus nerve stimulation led to a significant increase in sFC within the anterior cingulate gyrus. In a separate study, Sun et al. (2020) revealed that divergent thinking training intervention resulted in a significant increase in sFC within the anterior cingulate gyrus. Similarly, we found a higher sFC between the bilateral V1 and L-ACG in the present study. Our findings suggest that patients with HM have difficulty switching between brain networks, which leads to impairments in cognitive and emotional processing functions. Furthermore, our findings suggest that neural hyperactivity between the bilateral V1 and L-ACG is a compensatory response to the loss of cognitive and emotional processing functions in HM patients.

Additionally, we discovered that patients with HM had considerably greater sFC values between the left V1 and L-SPG. The superior parietal gyrus is a component of the apical region of the parietal lobe, surrounded by the postcentral gyrus, precuneus, and inferior parietal lobules of the parietal lobe. The superior parietal gyrus is within the anatomical area of the dorsal attention network (He et al., 2007), which participates in top-down control of visual attention (Rajan et al., 2021). As a brain network that focuses human attention, the dorsal attention network directs attention to the most salient and active brain networks. Previous studies have shown that the dorsal attention network maintains top-down attention control, allowing focus toward and away from external noise or environmental changes; conversely, the ventral attention network interrupts ongoing cognitive activities (Corbetta et al., 2008) and causes changes *via* bottom-up attention control (Corbetta and Shulman, 2002). Zou et al. (2021) found that the sFC of the superior parietal gyrus was increased in patients with chronic migraine, suggesting that the saliency of painful input increases in response to aural stimuli. Li et al. (2014) revealed that patients with primary insomnia exhibit significantly increased sFC in the superior parietal gyrus, thereby offering additional insights into the neurobiological mechanism of working memory deficiency caused by primary insomnia. Ye et al. (2019) reported that the white matter hyperintensities (WMH) with cognitive impairment (CI) group led to a significant increase in sFC within the superior parietal gyrus, which may reflect a compensatory functional enhancement. Furthermore, Huang et al. (2019) demonstrated that comatose patients had significantly increased sFC values in the superior parietal gyrus, implying that an increased sFC is associated with compensatory remodeling. Consistent with the previous findings, we demonstrated that patients with HM had significantly increased sFC values between the left V1 and L-SPG. Thus, patients with HM may have an inability to focus their attention to the network that is currently most active, leading to impaired top-down control of visual attention. Additionally, hyperactive neuronal connections between the left V1 and L-SPG may be a compensatory mechanism that protects against the loss of top-down control of visual attention in HM patients.

### 4.2. dFC alterations and their significance

The calcarine cortex and lingual gyrus are anatomical regions in the V1 that receive visual signals from the visual pathway, then transmit those signals to the higher visual cortex (Beckmann et al., 2005). These rich dynamic properties exhibited by early visual neurons suggest that V1 does not encode the environment in a static manner; it exhibits rich spatial and temporal dynamic features (Lazar et al., 2021). Huang et al. (2018b) found that amplitude of low-frequency fluctuation values in the bilateral lingual gyrus were considerably lower in retinitis pigmentosa patients than in HCs. Tong et al. (2021) demonstrated that patients with iridocyclitis displayed significantly lower FC between the V1 and both the bilateral calcarine. Dan et al. (2019) revealed that regional homogeneity values in the lingual gyrus were significantly lower in retinitis pigmentosa patients than in HCs. Furthermore, Wen et al. (2018) also found that late blindness patients showed a decreased FC between the V1 and the bilateral calcarine cortex/lingual gyrus. Additionally, Huang et al. (2020) demonstrated that the dynamic amplitude of low-frequency fluctuation/dFC of the bilateral calcarine cortex/lingual gyrus was lower in patients with late blindness than in HCs. Consistent with the previous findings, the present study showed that HM patients exhibited significantly decreased dFC values between the L-CAL and left V1 and the R-LING and right V1. The dFC is an indicator of the degree of spontaneous neural activity, which represents temporal variation in energy consumption and reflects neural network adaptability. Compared with sFC, dFC can better reflect the dynamic involvement of different brain regions in the actual brain and is considered a more accurate representation of functional brain networks (Zhao et al., 2020). Therefore, when the dFC value between the L-CAL and left V1 and the R-LING and right V1 in HM patients decreases significantly, it may be difficult to accept visual stimulation. This leads to impairment of visual information processing functions.

This study had some limitations. First, the number of HM patients was limited. Second, the data were frequently affected by unavoidable factors in the fMRI environment (e.g., heartbeat, muscle beat, and respiratory motion). Third, there remains debate regarding the selection of sliding window length because no standardized criteria have been established. An inadequate window length results in insufficient time points in each window to generate a stable dFC, but an excessive window length may reduce temporal variation in FC and thus fail to detect valid connections. Finally, the duration of HM varied among patients, which may have impacted the reliability of the results. In future studies, we plan to increase the sample size, improve the testing environment, and expand the size of the sliding window.

## 5. Conclusion

The results of this study indicated that, compared with HCs, patients with HM have altered sFC and dFC values in various brain regions, implying that HM causes extensive changes in static and dynamic spontaneous brain activity; these changes presumably lead to the corresponding clinical manifestations. Our findings improve the broader understanding of altered neural mechanisms in HM patients and provide new insights into potential neural causes of vision loss in those patients.

## Data availability statement

The original contributions presented in the study are included in the article/supplementary material, further inquiries can be directed to the corresponding author.

## Ethics statement

The studies involving human participants were reviewed and approved by the Nanchang University’s First Affiliated Hospital’s Medical Ethics Committee (Jiangxi Province, China). The patients/participants provided their written informed consent to participate in this study. Written informed consent was obtained from the individual(s) for the publication of any potentially identifiable images or data included in this article.

## Author contributions

YJ was responsible for writing the manuscript. S-QH was in charge of proofreading and refining the manuscript’s wording. QC, W-WF, P-PZ, X-LC, B-LS, BW, and Q-YH contributed to data collection and statistical analyses. YJ and S-QH designed the protocol and contributed to the MRI analysis. YJ, S-QH, and X-RW designed the study, oversaw all clinical aspects of study conduct, and prepared the manuscript. All authors contributed to the article and approved the submitted version.
